# The SNP rs10911021 is associated with oxidative stress in coronary heart disease patients from Pakistan

**DOI:** 10.1186/s12944-017-0654-8

**Published:** 2018-01-05

**Authors:** Saleem Ullah Shahid, Shabana, Steve Humphries

**Affiliations:** 1 Department of Microbiology and Molecular Genetics, University of the Punjab, Lahore, 54590 Pakistan; 2 Centre for Cardiovascular Genetics, British Heart Foundation Laboratories, University College London, WC1E6JF, London, UK

**Keywords:** Oxidative stress, MDA, GSH, GSSG

## Abstract

**Background:**

rs10911021 (a single nucleotide polymorphism present upstream of the *GLUL* gene) affects glutamic acid metabolism, and was shown to be associated with coronary heart disease (CHD) in patients with T2DM but a definite mechanism is unknown. It may affect glutathione cycle, an important effector in the antioxidant defense mechanism, in the cells. We checked the association of this SNP with CHD and oxidative stress biomarkers, malondialdeheyde (MDA), GSH and GSSG in Pakistani patients.

**Methods:**

A total of 650 subjects (425 CHD cases and 225 controls) were genotyped by TaqMan allelic discrimination technique. The levels of MDA, GSH and GSSG were measured by standard protocols.

**Results:**

The risk allele frequency was higher in cases than controls, but the difference was insignificant (*p* = 0.55). The SNP was not associated with CHD (*p* = 0.053) but when the analysis was limited to CHD patients having DM, a significant association (*p* = 0.03) was observed. The blood levels of MDA and GSSG were higher while that of GSH was significantly lower in the cases than the controls (*p* < 0.05). Each risk allele increased MDA and GSSG by 0.29 (0.036) mmol/l and 0.4 (0.04) mmol/l, respectively, while decreased GSH by −0.36 (0.03) mmol/l. The SNP was not associated with any of the tested blood lipids.

**Conclusion:**

The SNP rs10911021 was associated with CHD only in patients having diabetes, but the SNP was associated with total oxidative stress biomarkers MDA and GSH and GSSG levels. As the SNP rs10911021 showed significant association with oxidative stress parameters and these parameters should an increased oxidative stress in the CHD subjects, it can be concluded that the SNP may have contributed to increase the risk of heart diseases in the diabetic subjects by increasing the oxidative stress.

## Background

Oxidative stress results from an imbalance between the production of oxidants and their clearance by antioxidant defence mechanism [1]. The reactive oxygen species (ROS) are free radicals produced during aerobic respiration and being highly unstable, react with certain cell components like lipids, proteins and nucleic acids [2, 3]. Body has antioxidant defence mechanism consisting of antioxidant molecules like glutathione and enzymes like super oxide dismutase, glutathione reductase, catalase and glutathione peroxidase to cope up with oxidative stress. Among the biomolecules, lipids are most vulnerable to oxidative stress. Lipids such as membrane polyunsaturated fatty acids and low-density lipoprotein cholesterol (LDL-C) are main targets. Lipid peroxidation leads to formation of lipid hydroperoxides which breakdown to aldehydes through a chain of reaction [4]. Aldehydes are relatively stable compared to the free radicals and can diffuse from their site of synthesis to react with other cell components [5].

The direct approach for the determination of oxidative stress is either the measurement of free radicals or the extent of lipid peroxidation. The lipid peroxidation cannot be measured by the concentration of primary products (hydroperoxides) because of the labile nature of free radicals which may be ROS or hydroperoxides. Consequently, the lipid peroxidation determination relies on the indirect approach which is the measurement of secondary end products, aldehydes [6]. The most commonly used biomarker as an index of lipid peroxidation is Malondialdehyde (MDA), a naturally occurring relatively stable and low molecular weight end product of membrane lipids peroxidation produced along other byproducts [7]. MDA itself is a colourless end product which is measured in terms of thiobarbituric acid reactive substances (TBARS) through the thiobarbituric acid (TBA) test.

The oxidative stress can also be estimated by the status of antioxidant system in body. Glutathione, a tripeptide consisting of cysteine, glutamic acid and glycine is a ubiquitous molecule in living system. Reduced glutathione (GSH) is present mainly in free form and is converted to oxidized form (GSSG) by enzyme GSH peroxidase during the oxidative stress. The oxidized form is converted back to reduced form by the enzyme GSSG reductase. The synthesis and breakdown of glutathione takes place by the ɣ- glutamyle reaction. The glutathione cycle with its associated enzymes, glutathione peroxidase and glutathione reductase serves to maintain the overall oxidative milieu of the cell [8]. The redox status of the cell depends upon the relative concentrations of GSH and GSSG [9]. Because glutathione protects the cell from oxidative stress, its availability in reduced form is mandatory to control the redox status of the cell. Because blood glutathione level is an index of glutathione status in other less accessible tissues, red blood cell glutathione is considered to represent whole body glutathione status [10].

There is growing evidence that oxidative stress causes many chronic ailments like aging, diabetes [11], Alzheimer’s disease, infertility [12] and cardiovascular diseases [13]. Many studies have investigated the causal link between oxidative stress and CHD as the administration of antioxidants relived from the cardiac cell injury [14–16].

The SNP rs10911021 is an intergenic C/T variant, located nearby glutamate-amonia ligase (*GLUL*) gene and was found to downregulate the gene [17]. The *GLUL* is located at 1q31 and encodes glutamate ammonia ligase (glutamine synthetase) enzyme involved in glutathione biosynthesis. The enzyme inefficiency (deficiency or impaired function) may cause deficiency of glutamine and glutathione synthetase. Glutamine is an abundant aminoacid used in the biosynthesis of other aminoacids and glutathione [17].

The SNP rs10911021 which affects glutamic acid metabolism was reported to be associated with coronary heart disease (CHD) in subjects having type 2 diabetes mellitus (T2DM) although no definite mechanism was proposed [17, 18]. We proposed that the risk allele may cause CHD due to oxidative stress by interfering with the intermediates of glutathione cycle. The objective of this study was to investigate the effect of this SNP on oxidation status of the body. For this purpose GSH and GSSG levels were measured as indicators of oxidation status and the lipid oxidation end product, MDA, was used as an index of oxidation stress.

## Methods

### Study subjects

The study consisted of 425 CHD patients and 225 controls as described previously [19]. The CHD cases were ischemic heart disease patients diagnosed on the bases of ECG, cardiac echo, radiological and biochemical reports. All cases were recently diagnosed, not taking any lipid lowering or anti-hypertensive drugs. Controls were age and sex matched individuals without any history of cardiovascular disease. Among CHD patient, 275 had type 2 diabetes and among controls, 30 subjects were diabetic. The participants gave a written consent and the study was approved by the institutional ethical committee, University of the Punjab, Lahore.

### Determination of GSH and GSSG

The red cell GSH and GSSG were measured from red cell haemolysate, prepared from fresh blood. Briefly, 300 μl of well mixed fresh whole blood was mixed with double amount of cold sterilized normal saline and centrifuged at 4000 g for 2 min. Supernatant was discarded and red cell pellet was resuspended in the residual fluid. The cells were washed thrice in the same way with normal saline. A 500 μl of ice cold autoclaved distilled water was added to washed RBCs and mixed with the help of vortex mixer. The lysate was centrifuged at 10,000 g for 5 min to pellet down RBC membranes (ghost cells). The lysate was put on ice during subsequent analyses.

Red cell GSH and GSSG levels were measured using Ellman’s reagent by the reduction of 5, 5ˊ- dithiobis-2-nitrobenzoic acid (DNTB) as described previously [20]. The reaction buffer consisted of 0.1 M PO_4_ buffer (pH 7), 0.5 mM EDTA and low concentration of DNTB (3 mM) in absolute ethanol. A 10 mM nicotineamide dinucleotide phosphate hydrogen (NADPH) and 1 unit of glutathione reductase was used. All the reagents and enzyme was purchased from Sigma Aldrich (USA).

### Determination of malondialdehyde

The MDA concentration was measured as thiobarbituric acid reactive substances (TBARS) according to Ozturk et al. [21]. The method depends upon the precipitation of proteins, bilirubin, lipoproteins and other interfering substances by boiling in trichloroacetic acid (TCA). The assay mixture consisted of 10% TCA and 0.8% TBA. Butylated hydroxyl toluene (BHT) was used to reduce oxidation during the heating process.

### Genotyping rs10911021

Genomic DNA was extracted from peripheral blood using Promega Wizard® genomic DNA purification kit. The DNA was quantified using nanodrop (ND-8000, USA) and diluted to a standard concentration of 1.25 ng/μl. A 4 μl of this dilution was used to get the final working concentration of 5 ng of DNA. A liquid handling robotic system (Biomerk FX, Beckman Coulter) was used to spot DNA in 384 well plates (Micro Amp). After spotting the dilution was allowed to evaporate at room temperature to get DNA in dried form.

The genotyping was done by the TaqMan allelic discrimination technique. The reaction mixture consisted of 1X qPCR master mix (KAPABiosystems, USA), 100 nM of each primer, 100 nM of each probe, ROX high (0.4 μl/20 μl reaction mixture), 5 ng DNA in dried form and sigma water as required. The PCR program run on thermal cycler BioRad (C1000TM) consisted of an initial temperature of 50 °C for 2 min, denaturation/enzyme activation at 95 °C for 10 min followed by amplification for 40 cycles each cycle consisting of denaturation at 95 °C for 15 s and amplification at 60 °C for 1 min. After amplification the results were analyzed on ABI Prism 7900HT (Applied Biosystems/Life Technologies) and the genotypes were called using sequence detection software (SDS version 2.0). The genotypes were also confirmed randomly by conventional direct DNA sequencing (source biosciences, UK) to check the accuracy of technique and the results were concordant.

### Statistical analysis

Statistical analyses were performed using the Statistical Package for Social Sciences (SPSS version 22, IBM statistics). The study population was checked for Hardy Weinberg equilibrium (HWE) by Chi-square test. The student *t*-test was used to compare variables between case and control groups. Allele and genotype frequencies were calculated by an excel spreadsheet and chi-square test was used to compare allele frequencies between cases and controls. The association of genotypes with CHD was checked by binary logistic regression. The mean MDA, GSH and GSSG values for each genotype was calculated by ANOVA and their increase per allele was calculated by linear regression. An online power calculator (https://www.dssresearch.com/KnowledgeCenter/toolkitcalculators/statisticalpowercalculators.aspx) and G Power software were used for power analyses. A *p*-value <0.05 was considered a significance cutoff for all tests.

## Results

### Study subject characteristics

The general characteristics of the subjects included in the study are listed in Table 1. The proportion of males and females did not differ significantly among the participants. The mean age of cases (59.07 ± 12.66) and controls (55.97 ± 10.42) differed significantly (*p* = 0.002). The proportion of comorbidities, including diabetes, hypertension and obesity was higher in CHD cases than controls. The serum total cholesterol, LDL-C and triglyceride levels were higher and high density lipoprotein cholesterol (HDL-C) was lower in CHD cases than controls. The mean weight, BMI and WHR for obese subjects were 96.16 ± 15.95, 36.92 ± 6.46 and 1.00 ± 0.09 respectively, for CHD subjects were 67.13 ± 11.95, 22.46 ± 6.75 and 0.81 ± 0.06 respectively and for control subjects 67.13 ± 9.56, 21.46 ± 9.11 and 0.85 ± 0.10 respectively. The study power assuming a standard deviation of 1 and with a sample size of 650 was 81%.

**Table 1:** General characteristics of the study population, continuous variables are expressed as mean ± SD and categorical variables are expreesed as %

Variables	Cases (425)	Controls (225)	*p*-value
Gender (M/F)	250/175	122/103	0.271
Age (years)	59.11 ± 12.64	56.01 ± 10.44	0.002
Diabetes (%)	275 (64.6)	30 (13.6)	5.1 × 10^−34^
Hypertension (%)	264 (62.1)	37 (16.4)	8.9 × 10^−28^
TC	207.5 ± 53.65	175.4 ± 43	<0.001
LDL-C	105.95 ± 28.9	84.7 ± 16.9	<0.001
HDL-C	45.2 ± 11.9	67.4 ± 16.3	<0.001
TG	212.4 ± 70	188 ± 66.3	<0.001

*TC* total cholesterol, *LDL-C* low density lipoprotein cholesterol, *HDL-C* high density lipoprotein cholesterol, *TG* triglycerides, *p* value: level of statistical significance

### Allele and genotype frequencies of rs10911021

The allele and genotype frequencies of the rs10911021 variant are shown in Table 2. The risk allele (C) frequency (RAFs) in CAD group was higher than controls (0.678 vs 0.624), although the difference was not statistically significant (*p* = 0.055). Genotype distribution revealed following distribution among the cases and controls (CHD: CC = 45.3%, CT = 45%, TT = 9.7%; vs controls CC = 38.5%, CT = 48%, TT = 13.6%). The SNP was not associated with CHD, OR 1.3 (1–1.6. *p* = 0.05) but when the analysis was limited to diabetic subjects (275 T2DM patients out of 425 CHD patients) only, a significant association was observed (*p* = 0.03).

**Table 2:** Allele and Genotype Frequencies of rs10911021 in cases and controls

Allele/Genotype	Frequency (%)					
	Overall subjects			Diabetic Subjects only		
	Cases (425)	Controls (225)	*p*-value	Cases (275)	Controls (30)	*p*-value
TT	9.7	13.6	0.053	16.1	6.3	0.034
TC	45	48		36.7	33.3	
CC	45.3	38.5		47.2	60.4	
T	32.2	37.6		20.7	34.4	
C	67.8	62.4		79.3	65.6	

### Blood levels of oxidative stress biomarkers

To check the significance of difference of the blood levels of oxidative stress indicators between the cases and the controls, student *t*-test was used. A two tailed test showed that the mean MDA and GSSG were significantly higher while mean GSH was lower in cases than controls in cases than controls. The association of each parameter with CHD was checked using binary logistic regression and all these parameters were found to be associated with CHD (Table 3).

**Table 3:** comparison of MDA, GSH and GSSG among cases and controls and their CHD OR

Variables	Cases	Controls	*P* value	OR (CI)	*p*-value
TBARS mmol/l	3.15 ± 0.65	2.89 ± 0.51	4.2 × 10^−7^	2 (1.5–2.6)	<0.001
GSH mmol/l	5.4 ± 0.46	5.9 ± 0.48	6.7 × 10^−35^	0.8 (0.78–0.85)	<0.001
GSSG mmol/l	4.31 ± 0.62	3.80 ± 0.50	5.1 × 10^−24^	4.8 (3.4–6.8)	<0.001

*OR* odds ratio, *CI* confidence of interval

### Effect of rs10911021 polymorphism on oxidative stress biomarkers

A steady increase in the mean MDA and GSSG and a decrease in mean GSH from wild type homozygous to heterozygous to the homozygous polymorphic genotype was observed. Linear regression was employed to determine the per risk allele increase/decrease in the mean oxidative stress parameters. The *p*-values indicate that the presence of the risk allele is strongly associated with the increase in TBARS/GSSG and decrease in GSH in the studied subjects (Table 4).

**Table 4:** Effect of rs10911021 Genotypes on the mean values of oxidative stress indicators

Variables	TT	TC	CC	β (SE)	*p*-value
MDA mmol/l	2.59 ± 0.41	3.00 ± 0.59	3.23 ± 0.68	0.29 (0.036)	<0.001
GSH mmol/l	6.29 ± 0.23	5.6 ± 0.46	5.4 ± 0.5	−0.36 (0.03)	<0.001
GSSG mmol/l	3.62 ± 0.46	3.99 ± 0.51	4.40 ± 0.65	0.4 (0.04)	<0.001

β: is the effect size which is per allele increase/decrease in the variable, SE: standard error of mean. (The analysis has been done on the whole study cohort)

## Discussion

Serum thiobarbituric acid reactive substance (TBARS) measurement is a relatively nonspecific indicator of oxidative stress as the MDA is neither the sole end product of lipid peroxidation nor it is an exclusive product of fatty acid oxidation. MDA can also react with compounds other than TBA and MDA can also be produced from other compounds and during heating process while MDA measurement [22]. This phenomenon was addressed by the addition of BHT in the reaction mixture.

The variant rs10911021 is a new locus identified to be associated with diabetes in subjects with coronary heart disease [17]. Since its identification, only a few studies have investigated its role in different diseases. One study identified this variant to be a predictor of all cause mortality in diabetic subjects [18]. This intergenic SNP is approximately 270 Kb from the gene encoding glutamate ammonia ligase (*GLUL*) enzyme belonging to the glutamine synthase family. It has been found that individuals homozygous for the risk allele (C) have a lower plasma pyroglutamic acid/glutamic acid ratio resulting in impairment of the γ-glutamyl cycle which consequently increases oxidative predisposing diabetic individuals to CHD [17].

The GLUL also known as glutamine synthase catalyzes the conversion of glutamic acid (Glu) and ammonia into glutamine (Gln) [23]. Both Glu and Gln play important roles in human physiology. Glu is a key intermediate of the γ-glutamyl cycle which produces the anti-oxidant glutathione [24] while Gln regulates many important cell processes including cell proliferation, inhibition of apoptosis, and cell signalling [25]. Gln has been proved to be cardioprotective when administered enterally and paraenterally in many clinical trials [26, 27].

In the current study, we observed a directionally consistent association of the risk allele wth the diabetes in CHD patients. The minor allele showed to be the protective allele in the current study as well as previous studies. Although the observed association was not statistically significant, the power analysis showed that we had 81% power to detect the effect observed by Qi et al. Abnormal metabolism of glutamate and glutamine has been shown to be related to insulin resistance, type 2 diabetes, and cardiovascular diseases in many epidemiological studies [28–30]. The mechanism through which the changes in metabolism of these amino acids can lead to increased CHD risk is unclear at present. However, the proposed mechanisms are based on the understanding of alterations of glutamate and glutamine metabolism physiological pathways, the consequences that one would expect from the reduced GLUL expression. This in turn may affect glutathione cycle and the antioxidant defense mechanism in the cells.

We observed significantly different blood levels of MDA, GSH and GSSG between the cases and controls with GSH levels lower while that of MDA and GSSG higher in the cases compared to the controls indicating an overall oxidative stress in the CHD patients. It has been reported previously that decreased activity of antioxidant enzymes is associated with increased oxidative stress and vascular endothelium dysfunction in patient with chronic heart failure [31]. In addition, this oxidative stress affects other organs as well including pancreas ultimately causing islet damage leading to diabetes [32]. The increased oxidative stress in CHD patients can therefore lead to diabetes.

The SNP rs10911021 did not show association with the risk of type 2 diabetes in Diabetes Genetics Replication and Meta-analysis Consortium (DIAGRAM) study, which suggested that the association of the variant with CHD involves mechanism distinct from that in type 2 diabetes [33]. In addition, the variant did not show association with the serum fasting insulin or glucose levels, rejecting the hypothesis that insulin resistance can be the underlying mechanism [34]. However, it was associated with a lower plasma pyroglutamic-to-glutamic acid ratio, suggesting a possible mechanism for increase in CHD risk by lowering the serum level of the natural antioxidant glutathione. This association has also been replicated in the KORA/Twins UK metabolomic databases (*p* = 0.00096 in KORA (https://www.helmholtz-muenchen.de/ibis/research/metabolomics/projects/index.html). It should be noticed that additional pathways that are not directly related to glutamate and glutamine may also contribute to vascular biology and atherogenesis, e.g., a variety of lysophospholipids were found to be associated with rs10911021 in some databases [33, 35, 36]. In future, further studies are therefore needed to identify the mechanisms underlying the effect of this locus in the development and progression of heart issues.

The limitations of the study included a relatively small sample size and inability to include more biochemical and anthropometric measures in order to observe the real behaviour of the variant in the Pakistani ethnic group. The findings of the study should be therefore replicated in larger cohorts in the future with more biochemical parameter included in order to validate the results observed in the current investigation and find out new associations, if any, of the variant.

## Conclusion

In conclusion, the SNP rs10911021 was associated with CHD only in patients having DM, but the SNP was associated with total oxidation stress biomarker MDA and GSH and GSSG values. Therefore, rs10911021 can increase the risk of heart diseases in the diabetic subjects by increasing the oxidative stress in the endothelial system.

## Abbreviations

CHD: Coronary heart disease
DNTB: 5, 5ˊ- dithiobis-2-nitrobenzoic acid
*GLUL*: Glutamate-ammonia ligase
GSH: Reduced glutathione
GSSG: Oxidized form
LDL-C: Low density lipoprotein cholesterol
MDA: Malondialdehyde
NADPH: Nicotineamide dinucleotide phosphate hydrogen
SPSS: Statistical Package for Social Sciences
T2DM: Type 2 diabetes mellitus
TBARS: Thiobarbituric acid reactive substances
TCA: Trichloroacetic acid

## References

[1] Maritim A, Sanders R, Watkins J (2002). Diabetes, oxidative stress, and antioxidants: a review. J Biochem Mol Toxicol.

[2] Babovic S, Im MJ, Angel MF, Manson PN (1998). Role of reactive oxygen species in optic nerve compression injury: a preliminary study. Ann Plast Surg.

[3] Valenzuela A (1991). The biological significance of malondialdehyde determination in the assessment of tissue oxidative stress. Life Sci.

[4] Dianzani M, Barrera G. Pathology and physiology of lipid peroxidation and its carbonyl products. In: Álvarez S, Evelson P, editors. Free Radical Pathophysiology. Kerala: Transworld Research Network; 2008. p. 19-38. ISBN: 978-81-7895-311-3.

[5] Esterbauer H, Schaur RJ, Zollner H (1991). Chemistry and biochemistry of 4-hydroxynonenal, malonaldehyde and related aldehydes. Free Radic Biol Med.

[6] Pryor WA (1989). On the detection of lipid hydroperoxides in biological samples. Free Radic Biol Med.

[7] Horton A, Fairhurst S, Bus JS (1987). Lipid peroxidation and mechanisms of toxicity. CRC Crit Rev Toxicol.

[8] Meister A, Larsson A (1995). Glutathione synthetase deficiency and other disorders of the gamma-glutamyl cycle. The Metabolic and Molecular Bases of Inherited Disease, Volume 1.

[9] Chai Y-C, Ashraf SS, Rokutan K, Johnston R, Thomas JA (1994). S-thiolation of individual human neutrophil proteins including actin by stimulation of the respiratory burst: evidence against a role for glutathione disulfide. Arch Biochem Biophys.

[10] Reed DJ, Fariss MW (1984). Glutathione depletion and susceptibility. Pharmacol Rev.

[11] Oberley LW (1988). Free radicals and diabetes. Free Radic Biol Med.

[12] Ramalho-Santos J, Amaral S, Oliveira PJ (2008). Diabetes and the impairment of reproductive function: possible role of mitochondria and reactive oxygen species. Curr Diabetes Rev.

[13] Ergul A, Johansen JS, Strømhaug C, Harris AK, Hutchinson J, Tawfik A, Rahimi A, Rhim E, Wells B, Caldwell RW (2005). Vascular dysfunction of venous bypass conduits is mediated by reactive oxygen species in diabetes: role of endothelin-1. J Pharmacol Exp Ther.

[14] Miwa K, Igawa A, Nakagawa K, Hirai T, Inoue H (1999). Consumption of vitamin E in coronary circulation in patients with variant angina. Cardiovasc Res.

[15] Buffon A, Santini SA, Ramazzotti V, Rigattieri S, Liuzzo G, Biasucci LM, Crea F, Giardina B, Maseri A (2000). Large, sustained cardiac lipid peroxidation and reduced antioxidant capacity in the coronary circulation after brief episodes of myocardial ischemia. J Am Coll Cardiol.

[16] Singh RB, Niaz MA, Rastogi SS, Rastogi S (1996). Usefulness of antioxidant vitamins in suspected acute myocardial infarction (the Indian experiment of infarct survival-3). Am J Cardiol.

[17] Qi L, Qi Q, Prudente S, Mendonca C, Andreozzi F, Di Pietro N, Sturma M, Novelli V, Mannino GC, Formoso G (2013). Association between a genetic variant related to glutamic acid metabolism and coronary heart disease in individuals with type 2 diabetes. JAMA.

[18] Prudente S, Shah H, Bailetti D, Pezzolesi M, Buranasupkajorn P, Mercuri L, Mendonca C, De Cosmo S, Niewczas M, Trischitta V (2015). Genetic variant at the GLUL locus predicts all-cause mortality in patients with type 2 diabetes. Diabetes.

[19] Shahid SU, Cooper JA, Beaney KE, Li K, Rehman A, Humphries SE (2017). Genetic risk analysis of coronary artery disease in Pakistani subjects using a genetic risk score of 21 variants. Atherosclerosis.

[20] Rehman A, Anjum MS, Hasnain S (2010). Cadmium biosorption by yeast, Candida Tropicalis CBL-1, isolated from industrial wastewater. J Gen Appl Microbiol.

[21] Ozturk A, Baltaci AK, Mogulkoc R, Oztekin E, Sivrikaya A, Kurtoglu E, Kul A (2003). Effects of zinc deficiency and supplementation on malondialdehyde and glutathione levels in blood and tissues of rats performing swimming exercise. Biol Trace Elem Res.

[22] Forman HJ, Augusto O, Brigelius-Flohe R, Dennery PA, Kalyanaraman B, Ischiropoulos H, Mann GE, Radi R, Roberts LJ, Vina J (2015). Even free radicals should follow some rules: a guide to free radical research terminology and methodology. Free Radic Biol Med.

[23] Krebs HA (1935). Metabolism of amino-acids: the synthesis of glutamine from glutamic acid and ammonia, and the enzymic hydrolysis of glutamine in animal tissues. Biochem J.

[24] Meister A, Tate SS (1976). Glutathione and related γ-glutamyl compounds: biosynthesis and utilization. Annu Rev Biochem.

[25] DeBerardinis RJ, Cheng T (2010). Q’s next: the diverse functions of glutamine in metabolism, cell biology and cancer. Oncogene.

[26] Lomivorotov V, Efremov S, Shmirev V, Ponomarev D, Karaskov A (2011). Glutamine is cardioprotective in patients with ischemic heart disease following cardiopulmonary bypass. Heart Surg Forum.

[27] Sufit A, Weitzel LB, Hamiel C, Dauber I, Rooyackers O, Wischmeyer PE (2012). Pharmacologically dosed oral glutamine reduces myocardial injury in patients undergoing cardiac surgery a randomized pilot feasibility trial. J Parenter Enter Nutr.

[28] Cheng S, Rhee EP, Larson MG, Lewis GD, McCabe EL, Shen D, Palma MJ, Roberts LD, Dejam A, Souza AL (2012). Metabolite profiling identifies pathways associated with metabolic risk in humans. Circulation.

[29] Lauzier B, Vaillant F, Merlen C, Gélinas R, Bouchard B, Rivard M-E, Labarthe F, Dolinsky VW, Dyck JR, Allen BG (2013). Metabolic effects of glutamine on the heart: anaplerosis versus the hexosamine biosynthetic pathway. J Mol Cell Cardiol.

[30] Newsholme P, Krause M (2012). Nutritional regulation of insulin secretion: implications for diabetes. Clin Biochem Rev.

[31] Landmesser U, Spiekermann S, Dikalov S, Tatge H, Wilke R, Kohler C, Harrison DG, Hornig B, Drexler H (2002). Vascular oxidative stress and endothelial dysfunction in patients with chronic heart failure. Circulation.

[32] Stadler K. Oxidative Stress in Diabetes. In: Ahmad SI, editor. Diabetes. New York: Advances in Experimental Medicine and Biology. Springer; 2013. p. 272-87. 10.1007/978-1-4614-5441-0_21 23393685

[33] Bowden DW, Cox AJ (2013). Diabetes: Unravelling the enigma of T2DM and cardiovascular disease. Nat Rev Endocrinol.

[34] Suhre K, Shin S-Y, Petersen A-K, Mohney RP, Meredith D, Wägele B, Altmaier E, Deloukas P, Erdmann J, Grundberg E (2011). Human metabolic individuality in biomedical and pharmaceutical research. Nature.

[35] Schmitz G, Ruebsaamen K (2010). Metabolism and atherogenic disease association of lysophosphatidylcholine. Atherosclerosis.

[36] Von Schacky C (2000). n− 3 fatty acids and the prevention of coronary atherosclerosis. Am J Clin Nutr.

